# Exploring the Mesenchymal Stem Cell Secretome for Corneal Endothelial Proliferation

**DOI:** 10.1155/2020/5891393

**Published:** 2020-02-05

**Authors:** Bert Van den Bogerd, Nadia Zakaria, Steffi Matthyssen, Carina Koppen, Sorcha Ní Dhubhghaill

**Affiliations:** ^1^Antwerp Research Group for Ocular Science (ARGOS), Translational Neurosciences, Faculty of Medicine, University of Antwerp, Wilrijk, Belgium; ^2^Department of Ophthalmology, Antwerp University Hospital, Edegem, Belgium; ^3^Netherlands Institute for Innovative Ocular Surgery (NIIOS), Rotterdam, Netherlands

## Abstract

Ex vivo grown human corneal endothelial cells (HCEnC) are a new emerging treatment option to treat visually impaired patients aimed at alleviating the current global donor shortage. Expanding HCEnC is still challenging, and obtaining cells in sufficient quantities is a limiting factor. It is already known that conditioned medium obtained from bone marrow mesenchymal stem cells can stimulate the proliferation of endothelial cells. The aim of this study was to take this work a step further to identify some of the underlying factors responsible. We confirmed the stimulatory effect of the mesenchymal stem cell secretome seen previously and separated the exosomes from the soluble proteins using size exclusion chromatography. We demonstrated the presence of exosomes and soluble proteins in the early and late fractions, respectively, with transmission electron microscopy and protein assays. Proliferation studies demonstrated that growth stimulation could be reproduced with the later protein-rich fractions but not with the exosome-rich fraction. Antibody assays revealed the presence of the secreted proteins EGF, IGFBP2, and IGFBP6 in protein-high fractions, but the growth enhancement was not seen with purified protein formulations. In conclusion, we confirmed the stimulatory effect of stem cell-conditioned medium and have determined that the effect was attributable to the proteins rather than to the exosomes. We were not able to reproduce the growth stimulation, however, with the pure recombinant protein candidates tested. Specific identification of the underlying proteins using proteomics could render a bioactive protein that can be used for *ex vivo* expansion of cells or as an *in vivo* drug to treat early corneal endothelial damage.

## 1. Introduction

The corneal endothelium is the inner cell layer of the cornea and is responsible for maintaining the hydration and transparency of the cornea. The cells form a single monolayer with a characteristic hexagonal morphology and regulate electrolyte and water flow by a presumed pump-and-leak mechanism [1]. It is generally accepted that these cells do not have the capacity to divide in vivo, and as a result, the absolute number of human corneal endothelial cells (HCEnCs) only declines over time [2]. Surgical trauma, for example, induced during cataract surgery or specific diseases (e.g., Fuchs' dystrophy) can expedite this cell loss dramatically. When endothelial cell density falls to below a certain threshold (arbitrarily set at 500 cells/mm^2^), the remaining cells cannot fulfil their function, water passively enters the cornea resulting in corneal oedema. If this cannot be reversed, the patient will progress to bullous keratopathy, a condition characterized by reduced vision and pain.

Currently, the only way to treat these patients is through corneal endothelial transplantation, a well-established, very successful technique that accounts for around 40% of all corneal transplantations performed [3]. Unfortunately, access to these transplantations are currently restricted by a global donor shortage, lack of global logistic supply chains, and cornea banks. One possible strategy to overcome these issues is to tissue engineer an endothelial layer in the lab. This product would be composed of ex vivo grown HCEnCs on a suitable cell scaffold for transplantation [1, 4, 5]. Although the scaffold approach is the most commonly explored, cell suspension therapies have also been trialed in 11 patients [6, 7].

Regardless of the delivery method, it is still very difficult to expand HCEnCs to sufficiently high numbers required for regenerative medicine approaches. This difficulty had made the pursuit of a proliferation-inducing substance an area of very active research, and a number of successful candidates have been found in the last decade. ROCK inhibitor Y-27632, nuclear catenin p120, and p38 mitogen-activated protein kinase inhibitor have all shown promise as endothelial growth promoters though greater amounts of cell expansion are required before these therapies can make the mainstream [8–10].

When looking for new putative growth stimulants, mesenchymal stem cells (MSCs) are an interesting therapeutic option. It has previously been seen that while MSC transplantation did result in a beneficial effect on cardiomyocytes, it was due to a paracrine effect rather than actively participating in tissue regeneration through differentiation [11]. The observation sparked the idea of utilizing the “secretome” or proteinaceous secretions of the cells for tissue regeneration rather than expecting the cells themselves to regenerate the tissue [12]. This strategy has already been used to stimulate corneal endothelial cell growth through stem cells by means of a conditioned medium, i.e., culture medium from stem cells that have been growing in it for a defined period [13]. During culture, stem cells release an abundance of soluble proteins (growth factors and cytokines), extracellular matrix proteins, and a variety of extracellular vesicles. The latter group can be further subdivided in apoptotic bodies, microvesicles, and exosomes, depending on their size and biogenesis [12]. Exosomes especially, containing signalling molecules such as proteins, m(i)RNA, and lipids, have gained tremendous attention in the field of regenerative medicine. In fact, they are effective mediators of fundamental cell processes and have been implicated in, for instance, antigen presentation, cell survival, and proliferation [14, 15].

The specific expression profile of the secretome is strongly dependent on the culture environment, namely, the cell substrate, basal medium, and supplements [16]. While this fortified “conditioned” medium has already been shown to stimulate proliferation in corneal endothelial cells, the underlying components responsible for this effect are not known [13, 17–20]. From the point of drug regulation and patient safety, it is important to identify the bioactive compounds so that it can be refined and quantified [16]. That is why our aim was to pinpoint the exact compounds in the secretome of bone marrow-derived mesenchymal stem cells that stimulate corneal endothelial proliferation. Upon identification of such a compound (protein or exosomes), it could easily be implemented in current growth mediums for ex vivo expansion of endothelial cells to generate corneal endothelial cell sheets or could even be further developed as topical eye drops to stimulate endothelial wound healing such as ROCK inhibitors [21].

## 2. Materials and Methods

### 2.1. Cell Culture

Bone marrow-derived stem cells (BM MSC) were a kind gift provided by Professor Stefano Ferrari from the Veneto Eye Bank Foundation and were isolated from healthy donors with informed consent approved by the Ethical Committee of Azienda Ospedaliera Universitaria Integrata Verona. BM MSC were isolated from bone marrow aspirates through their ability to attach to culture flasks. Detailed description of the isolation and characterization of the BM MSC was performed according to the methods described by Krampera and colleagues [22]. The BM MSC were cultured in DMEM supplemented with 10% foetal bovine serum and amphotericin B and gentamycin (Life Technologies, Carlsbad, CA, USA), and medium was refreshed every other day. All experiments were run with a conditioned medium produced by two different donors and only cells up to passage 6 were used.

B4G12 human immortalized corneal endothelial cells were purchased from Deutsche Sammlung von Mikroorganismen und Zellkulturen (DSMZ, Braunschweig, Germany) and cultured according to their instructions with minor modifications. The B4G12 cells were seeded onto plastic coated with FNC coating mix (Athena Enzyme Systems, Baltimore, USA), and the growth medium consisted of Human Endothelial SFM supplemented with 10 ng/mL bFGF (Life Technologies, Carlsbad, CA, USA) without any antibiotics.

All culture medium was refreshed every other day, and cells were subcultured using Trypsin-EDTA 0.05% (Life Technologies, Carlsbad, CA, USA) and seeded according to the downstream assay.

### 2.2. Conditioning and Processing of Medium

The conditioned culture medium was produced by growing BM MSC to 80% confluency in standard growth medium described above. Cultures were then washed twice with phosphate buffered saline (PBS) 1× and grown in DMEM with 10% exosome-depleted foetal bovine serum (exoFBS) (Life Technologies, Carlsbad, CA, USA). The medium was conditioned for 24 hours, centrifuged at 3,000 × g for 15 minutes and filtered using 0.22 *μ*m^2^ polyethersulfone filters (Merck, Darmstadt, Germany). The conditioned medium obtained was stored at -80°C until required.

### 2.3. Coculture Proliferation Studies

Corneal endothelial proliferation in coculture with BM MSC was measured over time using the PrestoBlue assay (Life Technologies, Carlsbad, CA, USA) in a transwell system, in the lower compartment, 30,000 B4G12 wells seeded onto the FNC-coated surface of 24 well plate and cultured in the standard medium. In the upper compartment, 5,000 and 10,000 BM MSC were seeded onto a 0.4 *μ*m^2^ pore cell culture insert (Greiner Bio-One, Vilvoorde, Belgium) and grown in (i) DMEM with 10% normal, (ii) exosome-depleted FBS, and (iii) corneal endothelial culture medium. Experiments were performed in duplo for each donor.

The PrestoBlue proliferation assay (Life Technologies, Carlsbad, CA, USA) was performed in duplo for each donor starting the next day on the lower compartment for a period of eight days. The reagent was diluted as per the manufacturer's instructions, incubated for 30 minutes with the cells, and transferred to 96 well black OptiPlates (Perkin Elmer, Waltham, MA, USA). Absorbance measurements were repeated three times for every condition using the Wallac 1420 VICTOR^3^ microplate reader (Perkin Elmer, Waltham, MA, USA) and normalized to a negative control (PrestoBlue without cells). Endothelial cells grown in the presence of an empty culture insert were used as a negative control.

### 2.4. Biological Assays

Scratch wound assays were performed using the WoundMaker (Essen Bioscience, Hertfordshire, United Kingdom) for 96 well plate following the manufacturer's guidelines. Briefly, 75,000 B4G12 cells were seeded per well in dedicated 96 well ImageLock plates (Essenbio, Hertfordshire, United Kingdom) and let to adhere overnight. Next, the WoundMaker tool was used to make a consistent scratch with a width of 170 *μ*m per well and washed twice to remove dislodged cells. Phase-contrast images were taken every two hours automatically to monitor wound closure over time in the IncuCyte. The images were analysed with an internally trained algorithm to show relative wound density over time, a metric that measures the density of cells in the wound relative to the cell density in the outside of the wound area. The experiment was performed in quadruplicate with different concentrations of the conditioned medium.

For proliferation assays with protein preparations, B4G12 cells were seeded in a 96 well plate as described earlier and let to adhere. Human EGF, IGF-BP2, and IGF-BP6 were purchased from Peprotech (London, United Kingdom) and reconstituted in PBS 1×. The plate was monitored using the IncuCyte live cell imaging system. Proliferation was expressed either as the increase of cellular confluence in percentage over time or population doubling times (PDT). To obtain PDT, proliferation curves were fitted through an exponential growth curve *Y* = *Y*0^∗^exp(*k*^∗^*X*) (GraphPad Prism), and PDT were extracted through the formula ln(2)/K with *K* being the rate constant. PDT were compared statistically using a nonparametric Kruskal-Wallis test where *p* < 0.05 was deemed significant.

### 2.5. Size Exclusion Chromatography (SEC)

The conditioned medium was concentrated using Centricon Plus-70 centrifugal concentrators with 100 kDa cut-off regenerated cellulose membranes (Merck, Darmstadt, Germany). 65 mL of conditioned medium was used as starting volume and centrifuged for 3,000 × g for 25 minutes following a 1,000 × g spin for two minutes to recover the retentate.

Size exclusion chromatography was performed with qEVoriginal columns (iZon science, Oxford, United Kingdom) to separate soluble proteins from exosomes. The columns were equilibrated with PBS 1× on room temperature before use, and the obtained retentates were diluted to obtain a final volume of 500 *μ*L for standardization. Being void volume, the first five fractions were discarded, followed by collection of 25 consecutive fractions of 500 *μ*L in low protein binding collection tubes (Life Technologies, Carlsbad, CA, USA). For the proliferation studies, the fractions were further concentrated using Amicon Ultra protein concentrators with a 100 kDa cut-off membrane of regenerated cellulose (Merck, Darmstadt, Germany) for seven minutes at 3,000 × g. The retentates, i.e., the fraction that is retained in the filter, were recovered by spinning the filters in an upside-down orientation for two minutes at 1,000 × g and were divided over two wells (approximately 40 *μ*L). Conditions were performed in duplicate, and the same volume of PBS 1× was added to control conditions.

### 2.6. Protein Quantitation

The fractions obtained of the SEC were tested using a Bradford protein assay (Life Technologies, Carlsbad, CA, USA) to acquire protein elution profiles. Each sample was diluted 1 : 3 in PBS 1× and incubated for 10 minutes with the Bradford reagent (1 : 1) at room temperature. Measurements were performed in duplo in Costar 96 well plate (Sigma-Aldrich, St. Louis, USA) and repeated three times. OD values were normalized using a negative control and converted to concentrations (*μ*g/mL) using an albumin standard curve (*R*^2^ = 0.996) (Life Technologies, Carlsbad, CA, USA).

### 2.7. Transmission Electron Microscopy

The presence of exosomes in the early SEC fractions (6-10) of two donors was confirmed using transmission electron microscopy (TEM) performed according to previous published protocols [23, 24]. Briefly, three drops per sample were placed on Parafilm, covered by a nickel grid and incubated at room temperature for one hour to absorb the EVs. The grids were then rinsed three times with PBS 1× and five times with Ultrapure water. Next, the samples were fixed for ten minutes with 2% glutaraldehyde and washed five times with Ultrapure water. The grids were then covered with 2% uranyl acetate for 15 minutes, followed by incubation in 0.13% methyl cellulose (K5-8) and 0.4% uranyl acetate for ten minutes, and allowed to dry at room temperature. The obtained samples were imaged with a Tecnai G2 Spirit BioTWIN (FEI, Eindhoven, The Netherlands).

### 2.8. Antibody Array

Analysis of the protein profile was achieved by a human growth factor antibody array (ab134002, Abcam, Cambridge, United Kingdom). After blocking the membranes, SEC retentates (500 *μ*L) were diluted to 1 mL, incubated overnight at 4°C, and washed accordingly. The membranes were further incubated with biotin-conjugated anti-cytokines for five hours at room temperature while gentle shaking and aspirated. Subsequently, HRP-conjugated streptavidin was added overnight at 4°C. Chemiluminescent detection was performed according to the instructions, and images were captured with the G:BOX (Syngene, Cambridge, United Kingdom) with cumulative exposure times of 5 sec, 30 sec, 1 min, 2 min, and 5 min.

### 2.9. Data Analysis

Data processing was performed using Microsoft Excel and statistical analysis with GraphPad Prism 6.0. Data are expressed as mean ± SD. After data exploration, data were tested for normality according to the sample size, Kolmogorov–Smirnov test, and histograms. In all cases, a nonparametric Kruskal-Wallis test was performed to check for a significant difference, except for the coculture studies, a Friedman test was used. *p* value < 0.05 was considered the threshold for significance.

## 3. Results

### 3.1. The Proliferative Effect of BM MSC-Conditioned Medium

**Figure 1:**
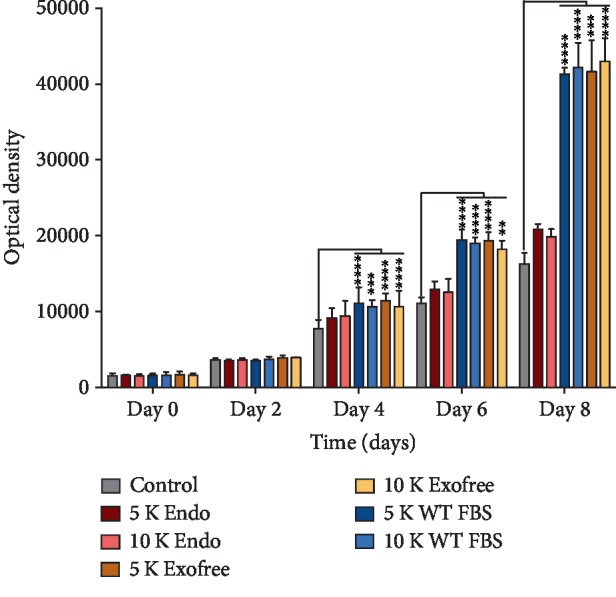
Corneal endothelial cell proliferation is indirectly stimulated when cocultured with BM MSC when they are grown in standard culture media. Only significant difference with the control condition are indicated. Endo: corneal endothelium medium; Exofree: BM MSC medium with exosome depleted FBS; WT FBS: BM MSC medium with wild-type FBS.

BM MSC directly stimulate proliferation B4G12 endothelial cells in a coculture set-up, while having different growth medium compositions of the upper compartment for the stem cells: endothelial cell medium (ENDO) vs. standard BM MSC medium with wild-type FBS (WT FBS) vs. standard medium with exosome-depleted FBS (Exofree). Cell proliferation was significantly increased in every experimental condition except when BM MSC were cultured in endothelial cell medium (Figure 1). There was no difference in response seen with different amounts of stem cells (5,000 or 10,000 BM MSCs) in the upper compartment on their proliferative effect.

**Figure 2:**
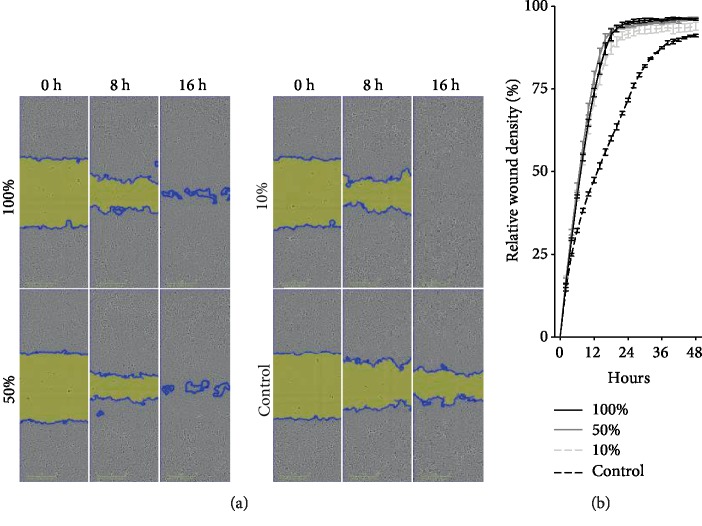
(a) Illustrative phase-contrast images of closing wound in vitro. (b) Graphic representation relative wound density over time. Relative wound density corrects for proliferation in the nonscratched areas.

Additionally, BM MSC exerted this effect also indirectly by adding a conditioned medium in different concentrations to endothelial cell cultures being 100%, 50%, and 10%, in a scratch wound assay. The wounds were >90% closed after 18 hours in each of the experimental conditions, while the control displayed only 60% closure (Figures 2(a) and 2(b)).

### 3.2. Exploration of the Conditioned Medium

**Figure 3:**
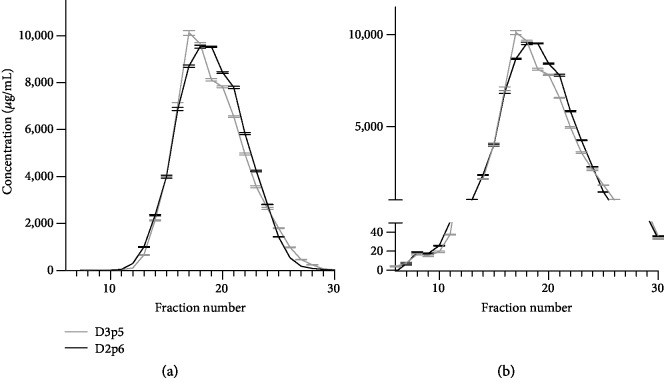
(a) Protein profile of the eluted fractions of two BM MSC donors with the highest protein profiles found around fraction 18. (b) When looking in detail to the earliest fractions, we found an elevation in protein concentration indicating the presence of exosomes.

In order to understand which secreted component of the BM MSCs caused this effect, we sought to separate different classes within the secretome, i.e., extracellular vesicles and secreted proteins through size exclusion chromatography. Following this, a Bradford assay revealed the protein profile of the fractions which showed the highest concentration of proteins in fractions 17 and 18 (Figure 3(a)), depending on the donor, with maxima reaching 10,000 *μ*g/mL. Protein concentration in a standard conditioned medium was around 40 *μ*g/mL (data not shown).

**Figure 4:**
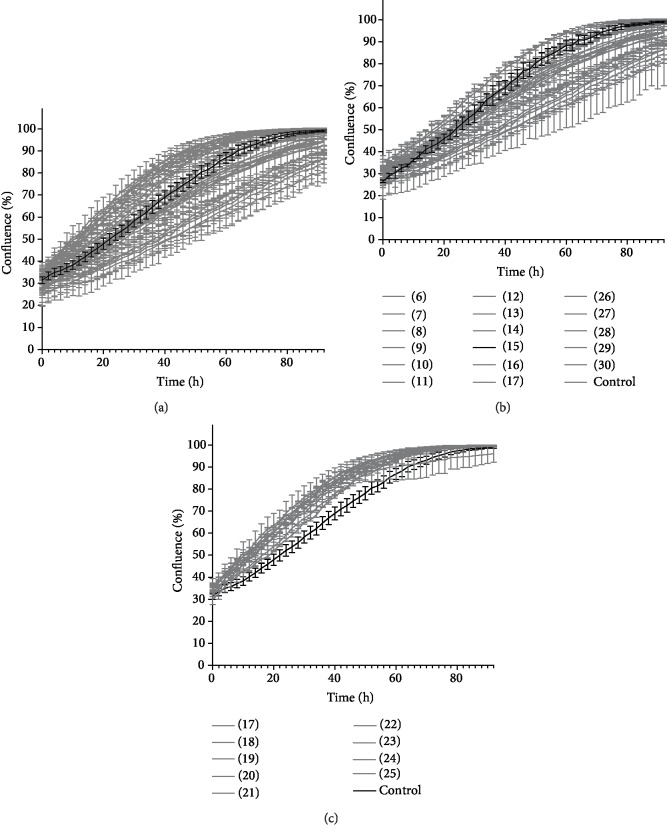
Proliferation curves of B4G12 cells supplemented with different fractions isolated by SEC with the control condition highlighted in black. (a) Proliferation curves over time of all fractions (#6-30). (b) Those who do not or negatively influence corneal endothelial growth (#6-16 and #26-30). (c) Those who stimulate cell proliferation.

There was also a slight increase in protein concentration in the early fractions (±20 *μ*g/mL) following the elution of the void volume (fractions 1-5), indicating the presence of exosomes indirectly by measuring their membranous proteins through a Bradford assay as an indirect quantitation of exosomes (Figure 3(b)). The separated fractions were further concentrated and used for individual proliferation assays (Figure 4(a)). Their results show that the fractions with a high protein concentration show a positive effect on cell proliferation (fractions 18-25) compared to the control (black line) (Figure 4(b)). On the other hand, the remaining fractions, including the ones with exosomes (fractions 7-9) did not stimulate endothelial growth compared to the negative control (Figure 4(c)).

### 3.3. Pinpointing the Content of the Fractions

Based on the obtained protein profiles, exosomes are expected in the early eluted SEC fractions and proteins, which have a longer retention time due to their smaller size, in the later fractions (Figures 3(a) and 3(b)). In order to confirm this, TEM and protein assays were performed on the early and late fractions, respectively.

**Figure 5:**
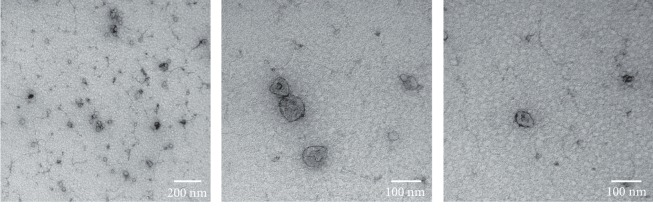
Three illustrative photos of exosomes captured using transmission electron microscopy. The vesicles are within the expected size range and display the characteristic central depression as a result from the TEM sample preparation.

TEM revealed the presence of exosomes in fractions 6 to 10 of both donors, however to a relatively low degree. The detected membranous vesicles showed a mean diameter of 61.91 ± 3.77 nm with a range of 23 nm-171 nm. The exosomes appeared rounded in shape and displayed a small depression centrally which is characteristic for exosomes that are observed with TEM (Figure 5).

**Figure 6:**
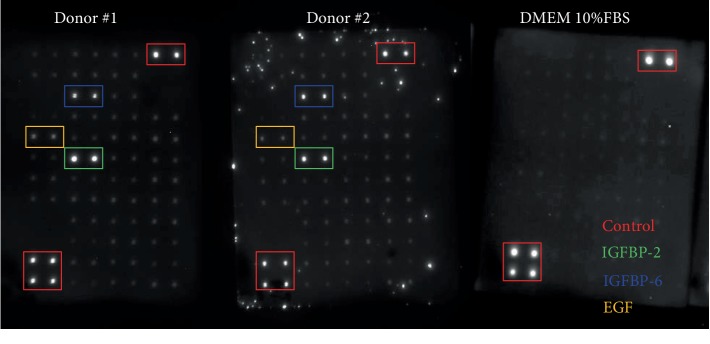
Growth factor assay reveals a positive signal for EGF, IGFBP2, and IGFBP6 for both donors. In the negative control, there was no signal for those growth factors. Donor #2 shows artefacts next to the blotted growth factors' antibodies.

Growth factor antibody arrays were performed on the samples that stimulated proliferation the most, namely, fractions 18 to 23, to identify secreted proteins. The full panel of growth factors that have been tested is displayed in supplementary [Supplementary-material supplementary-material-1]. It was observed that there was a positive signal in duplicate for epidermal growth factor (EGF), insulin-like growth factor binding proteins 2 and 6, IGF-BP2, and IGF-BP6, respectively (Figure 6). No positive signal was observed in the negative control, being basal MSC growth medium with 10% FBS.

### 3.4. Growth Factor Titration

**Figure 7:**
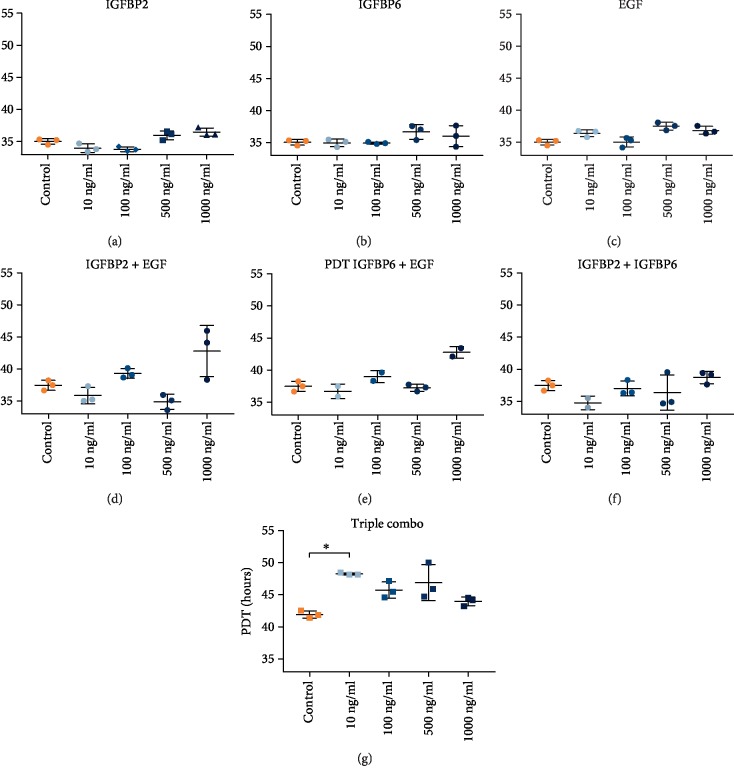
Comparison of population doubling times (PDT) in decreasing concentrations of single protein formulations (a–c), double growth factor combinations (d–f), and triple combination (g).

Additional proliferation studies were then carried out with purified formulations of the identified proteins in decreasing concentrations (range: 1000–10 ng/mL). Population doubling times (PDT) were calculated from the exponential growth curve and compared statistically. When performing proliferation assays with the individual formulations of the three identified growth factors, there was no stimulation of cell growth (Figures 7(a)–7(c)). In the next step, growth factors were combined in order to mimic the conditioned medium, step by step. When titrating the growth factors two by two, there was no significant decrease in PDT observed (Figures 7(d)–7(f)). In the combination with all three identified growth factors, there was neither a simulation of growth, even more, there was an increase in PDT, i.e., a slower cell growth, for cell supplemented with a 10 ng/mL combination of EGF-IGFBP2-IGFBP6 (Figure 7(g)).

## 4. Discussion

In this study, we confirmed that BM MSC-conditioned medium is capable of stimulating proliferation of immortalized corneal endothelial cells. We found that the stimulatory effect was due to the influence of secreted proteins rather than the exosome fraction. We also detected three growth factors that are specifically secreted in higher concentrations by BM MSC and tested their stimulatory effect with human purified proteins. Unfortunately, these assays did not reproduce the effect seen with the whole conditioned medium. Therefore, while they are present in the conditioned medium in high quantities, they are not responsible for the growth effects on their own. This raises the questions as to what fragment of the medium is responsible. It may be that the growth factor antibody array does not include the most relevant factor or that the responsible factor is detected but exerts its effect at a low concentration.

It is interesting that the epidermal growth factor (EGF) was identified in the conditioned medium that stimulated growth of corneal endothelial cells, but it was not effective in stimulating cell growth with a purified formulation. In contrast to this finding, EGF has previously been shown to promote proliferation when used as a supplement for the primary corneal endothelial culture medium by several groups in a concentration range of 5-10 ng/mL for human and bovine corneal endothelial cells, despite having no effect on our cultures [25, 26]. One possible explanation could be that the immortalized corneal endothelial cells are not sensitive to EGF supplementation compared to primary cells.

Insulin-like growth factor binding proteins (IGFBP) 2 and 6 were also detected in the protein-rich fractions that caused endothelial cell proliferation. IGFBP is a family of IGF binding proteins consisting of six members, each displaying subtle structural differences leading to differential binding capacities and functions [27]. IGFBP can inhibit or potentiate effects on IGF-induced pathways by regulating the availability of soluble IGF subtypes in the blood and extracellular space.

Furthermore, we confirmed the presence of exosomes in the early eluted fractions using the SEC columns through a protein assay and TEM. Exosomes are the smallest class of extracellular vesicles of only 30-150 nm in diameter that are secreted by every cell in the human body. Initially, they were thought to have no essential function, but work in the past decade indicates a promising future [28]. In our study, however, we did not manage to see any exosome-related effect on the cell cultures. Since isolation of exosomes is a rigorous task, chances are that we are not yet capable of isolating exosomes in sufficient concentrations. However, we would like to underline that it is possible that the capacity of exosomes or the robustness of isolation is overestimated since this is a relative new field that is gaining tremendous popularity, leading to a high publication rate and hasty conclusions [29]. For instance, the current golden standard of exosome isolation is still differential ultracentrifugation which is prone to both protein and nucleic acid contamination. Therefore, beneficial effects are possibly too often attributed solely to exosomes when there are still soluble proteins present. Here, we isolated and separated exosomes and proteins within the conditioned medium using SEC and demonstrated the role of proteins rather than exosomes in stimulating cell growth.

In this study, we included a limited number of donors from only one tissue source; however, it has been proven that MSCs isolated from other tissues can present a different secretome [30]. Therefore, it is interesting whether the MSC-conditioned medium from other tissue sources produces a similar beneficial effect or another set of growth factors. Additionally, in order to home in on the relevant protein, more advanced proteomic analysis techniques could also be implemented such as liquid chromatography and mass spectrometry [31]. However, such experiments would result in the identification of thousands of proteins, making it impossible to test separately and in combinations. Although we observed an effect of conditioned medium on the immortalized cells, the purified proteins had no effect on them, but we cannot simply extrapolate this to primary endothelial cells without further research.

## 5. Conclusions

A stem cell-conditioned medium can stimulate corneal endothelial cell medium. In detail, we have found that it is rather the proteins than the exosomes that account for this growth stimulation. However, we could not replicate this effect with pure recombinant proteins that were detected in the conditioned medium. In the future, finding the underlying responsible protein (combination) could be used for the production of a corneal endothelial cell therapy or applied as an in vivo drug for mild corneal endothelial dysfunction.

## Data Availability

The data used to support the findings of this study are available from the corresponding author upon request.

## Supplementary Materials

Supplementary 1 supplementary table 1: a plate map of all the growth factors that were examined for their presence in the fractions 18-23.

Supplementary 2 supplementary video 1: a time lapse showing the closure of the central wound in the presence of 0% (control), 10%, 50%, and 100% of the conditioned medium.
