# Supplementary material for: Exploring the Mesenchymal Stem Cell Secretome for Corneal Endothelial Proliferation
Source: Stem Cells Int. 2020 Feb 5;2020:5891393. doi: 10.1155/2020/5891393 (PMC7025074; doi:10.1155/2020/5891393)
Supplement: Supplementary 1 — supplementary table 1: a plate map of all the growth factors that were examined for their presence in the fractions 18-23. [file 5891393.f1.pdf]

.

Supplementary material

	A	B	C	D	E	F	G	H	I	J	K	L
1	Pos	Pos	Neg	Neg	AR	bFGF	b-NGF	EGF	EGF-R	FGF-4	FGF-6	FGF-7
2	Pos	Pos	Neg	Neg	AR	bFGF	b-NGF	EGF	EGF-R	FGF-4	FGF-6	FGF-7
3	GCSF	GDNF	GM-CSF	HB-EGF	HGF	IGFBP-1	IGFBP-2	IGFBP-3	IGFBP-4	IGFBP-6	IGF-1	IGF-1 SR
4	GCSF	GDNF	GM-CSF	HB-EGF	HGF	IGFBP-1	IGFBP-2	IGFBP-3	IGFBP-4	IGFBP-6	IGF-I	IGF-I SR
5	IGF-II	M-CSF	M-CSF-R	NT-3	NT-4	PDGF-Rα	PDGF-Rβ	PDGF-AA	PDGF-AB	PDGF-BB	PIGF	SCF
6	IGF-II	M-CSF	M-CSF-R	NT-3	NT-4	PDGF-Rα	PDGF-Rβ	PDGF-AA	PDGF-AB	PDGF-BB	PIGF	SCF
7	SCF-R	TGF-α	TGF-β	TGF-β2	TGF-β3	VEGF	VEGF-R2	VEGF-R3	VEGF-D	Blank	Blank	Pos

Supplementary table 1: The full panel of growth factors that have been tested.
